# The Dynamic Effects of Transposon Insertions on *AuxRP1* Transcription During Vegetative Development in *Zea mays*

**DOI:** 10.17912/micropub.biology.001640

**Published:** 2025-07-14

**Authors:** Julia M. Troy, Disha Bhalla, Kayla M. Cadet, Angeline G. Dishmey, Nathaly M. Hernandez, Zuhair Ishraq, Chloé Marchand, Spyro Markoulis, Resa B. Nelson, Nicholas Xhindolli, Dafang F. Wang

**Affiliations:** 1 Biology, Hofstra University, Hempstead, New York, United States

## Abstract

The *AuxRP1* gene ( *Zm00001eb053610* ) is involved in the auxin signaling pathway and stalk rot resistance in *Zea mays* . In this study, we examined four transposon insertion lines (UFMu-03429, UFMu-00414, UFMu-08200, and AcDs-00676) targeting *AuxRP1* . Transcription of *AuxRP1* of the insertion lines either decreased or remained unchanged at the juvenile (V3) stage but increased significantly at the transition/adult (V6) stage. We also analyzed its upstream gene *TSB2C* and downstream gene *Yucca2* . Our results show that transposon insertions can induce stage-specific changes in gene expression that affect related biosynthetic pathways.

**Figure 1. Relative expressions of the AuxRP1 and its pathway genes in transposon insertion lines:**
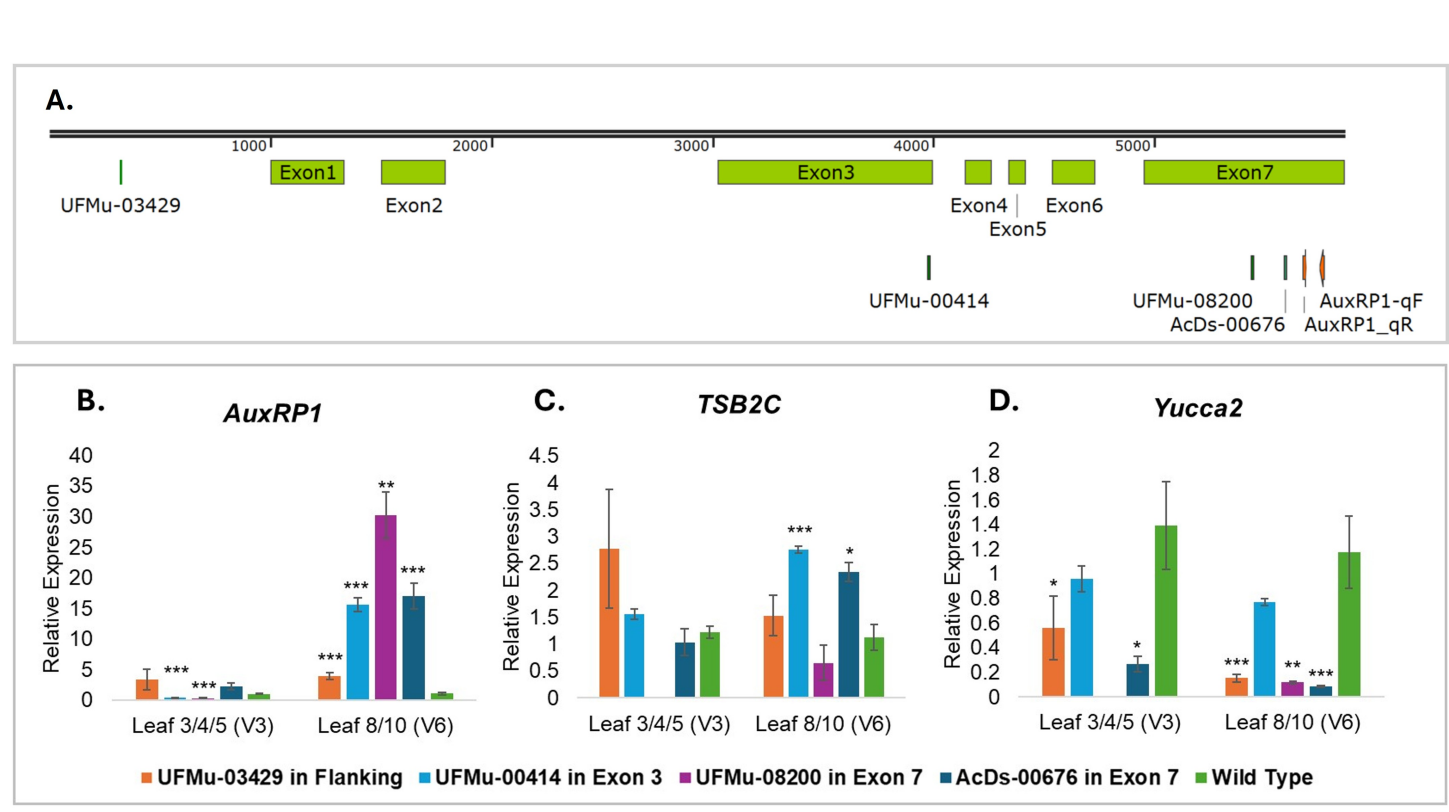
**A.** Gene model of *AuxRP1* ( *Zm00001eb053610* ) showing exons annotated in *Zm00001eb053610_T001* and transposon insertion sites for UFMu-03429 (flanking region), UFMu-00414 (exon 3), and UFMu-08200 and AcDs-00676 (exon 7). The orange arrows mark the positions of primers used in qRT-PCR for *AuxRP1.* **B–D.** Average relative transcript levels of *AuxRP1* , *TSB2C* , and *Yucca2* , respectively, in transposon insertion lines compared to wild-type (W22) plants at the juvenile stage (V3; leaves 3/4/5) and transition/adult stages (V6; leaves 8/10). Asterisks indicate statistical significance ( *p* < 0.05 as *, *p* < 0.01 as **, and *p* < 0.001 as ***) from the non-parametric Mann-Whitney U test on ∆Ct values (Chen et al., 2006). All data are based on two to three biological replicates and three technical replicates. Error bars indicate ± standard errors.

## Description

Transposable elements are mobile DNA sequences capable of moving within the genome, often disrupting gene function when inserted into coding or regulatory regions (Craig et al., 2002). They are widely used as tools of insertional mutagenesis to create knockout or knockdown mutants. In maize, which has a genome composed of approximately 85% transposable elements (Schnable et al., 2009; Stitzer et al., 2021), two mutagenesis systems are commonly used: Uniform *Mu* and *AcDs* . The UniformMu system was developed by introgressing active *Mu* elements into the W22 inbred background (McCarty et al., 2005), while the *AcDs* system, from W22-derived backgrounds, induces local mutagenesis through the mobilization of *Ds* elements (Sundaresan et al., 1995; Vollbrecht et al., 2010).

In this study, we used both the Uniform *Mu* and *AcDs* transposon systems to examine the transcriptional impact of insertions in the *AuxRP1* gene (Auxin Regulated Protein 1; *Zm00001eb053610* ). This gene contributes to stalk rot resistance by promoting indole-3-acetic acid (IAA) synthesis and suppressing benzoxazinoid production (Ye et al., 2019). We hypothesized that transposon insertions would reduce *AuxRP1* transcript levels during vegetative development. Four insertion lines were selected based on the *Zm00001eb053610_T001* annotation: one in the flanking region (UFMu-03429), one in exon 3 (UFMu-00414), and two independent insertions in exon 7 (AcDs-00676 and UFMu-08200). The plants tested for each line carried at least one copy of the insertion (see "PCR results for the insertion junctions in all the tested lines" in Extended Data); zygosity (homozygous or heterozygous) was not determined. *AuxRP1* transcript levels were measured in leaf tissues at two developmental stages: V3 (juvenile phase) and V6 (transition to adult phase).

At the V3 stage, we observed a significant decrease in *AuxRP1* transcription in the exon 3 (UFMu-00414) and exon 7 (UFMu-08200) lines [relative expression compared to W22: UFMu-00414: 0.43 ± 0.04, *p* < 0.001; UFMu-08200: 0.33 ± 0.07, *p* < 0.001], supporting our hypothesis. However, insertions in the flanking region (UFMu-03429) or exon 7 via *Ds* (AcDs-00676) showed no significant difference. However, when plants reached the V6 stage, marking the transition to the adult vegetative phase, we observed a surprising increase in *AuxRP1* transcription across all tested regions. This included the flanking region [UFMu-03429: 3.97 ± 0.61, *p* < 0.001], exon 3 [UFMu-00414: 15.61 ± 1.52, *p* < 0.001], and exon 7 [AcDs-00676: 17.06 ± 2.11, *p* < 0.001; UFMu-08200: 30.23 ± 3.78, *p* < 0.01]. The increase was more pronounced in the exon insertions compared to the flanking insertion. Additionally, all increases at the V6 stage were substantially greater than the decreases observed at V3.

To determine how changes in *AuxRP1* transcription influence other genes in the same biosynthetic pathway, we measured the transcript levels of *TSB2C* ( *Tryptophan synthase beta chain 2, chloroplastic;* *GRMZM2G005024,* an upstream gene involved in IAA synthesis) and *Yucca2* ( *Yucca2/YUCCA family monooxygenase;* *GRMZM2G159393* ) involved in the final step of IAA synthesis) (Ye et al., 2019). *TSB2C* transcription showed no significant changes at the V3 stage across all insertion lines. However, during the V6 stage, we observed increased expression in some insertion lines, including the exon 7 insertion [AcDs-00676: 2.34 ± 0.172, *p <* 0.05] and the exon 3 insertion [UFMu-00414: 2.76 ± 0.063, *p <* 0.001] (Fig. C). These increases were comparable to those observed for *AuxRP1* , though less pronounced in magnitude. In contrast to the consistent upregulation of *AuxRP1* and its upstream gene *TSB2C* , the downstream gene *Yucca2* showed a general decrease in transcription at both the V3 and V6 stages. This included reduced expression at the V3 stage in the flanking region insertion [UFMu-03429: 0.563 ± 0.260, *p <* 0.05] and exon 7 insertion [AcDs-00676: 0.267 ± 0.061, *p <* 0.05] (Fig. D). At the V6 stage, an even more pronounced decrease was observed in the flanking region [UFMu-03429: 0.15 ± 0.03, *p <* 0.001] and in exon 7 insertions [AcDs-00676: 0.087 ± 0.006, *p <* 0.001; UFMu-08200: 0.12 ± 0.008, *p <* 0.01].

In conclusion, our hypothesis that transposon insertions would reduce *AuxRP1* transcription is only supported at the V3 stage for insertions in exon regions. At this stage, the reduction in *AuxRP1* expression was accompanied by no change in its upstream gene *TSB2C* and a decrease in the downstream gene *Yucca2* . At the V6 stage, however, *AuxRP1* and *TSB2C* expression both increased, while *Yucca2* continued to show decreased expression. These results were inconsistent with our original hypothesis. In addition, the type of transposon ( *AcDs* or *Mu* ) did not seem to have a major effect on transcription levels in most cases. Instead, the location of the insertion, whether in an exon or flanking region, had a greater effect.

Despite the unexpected results at V6, we are confident in the reliability of our data. All experiments included two to three biological replicates and three technical replicates. The W22 inbred line was used as the wild-type control, and plant developmental stages were closely monitored. Each insertion line was tested independently by different student groups following a standardized protocol described in the “Methods” section.

One limitation of this study is the absence of data confirming whether the tested plants are homozygous or heterozygous for the insertions. Our results are only based on plants carrying at least one copy of the insertion, so they should be considered preliminary. These findings highlight the importance of experimentally verifying transcriptional effects when using insertion lines. A transposon insertion does not necessarily reduce gene expression at all developmental stages and should not be assumed to be a knockout or knockdown mutation without supporting evidence. Future studies should include phenotypic analyses, such as pathogen inoculation, to assess disease resistance associated with the insertions. We also plan to examine transcript structures in selected lines, which may help rule out alternative splicing as a cause of the unexpected transcriptional patterns.

## Methods

*Plant Growth*

Maize lines UFMu-03429, UFMu-00414, UFMu-08200, AcDs-00676, and the wildtype line W22 were obtained from the Maize Genetics Cooperation Stock Center. Seeds were germinated in small pots containing Premier B10281RG ProMix. At approximately the V2 developmental stage, seedlings were transplanted into larger pots to support continued shoot and root development. A diluted 10-10-10 (N-P-K) all-purpose fertilizer was applied at the time of transplanting and subsequently once per week to promote vegetative growth.

*Genotyping*

Genomic DNA was extracted from maize leaf tissue at the V1 developmental stage using the Quick-DNA™ Plant/Seed Miniprep Kit (Zymo Research D6020), following the manufacturer’s protocol. To identify plants carrying *Mu* transposon insertions in the *AuxRP1* gene, polymerase chain reaction (PCR) was conducted using PCR Master Mix (Sydlabs, MB067-EQ2B). Each reaction included a primer specific to the insertion flanking sequence and a primer from the *Mu* element (see “Reagents” for primer sequences). Tubulin (Zm00001d010275) primers were used as internal controls to assess DNA quality, while nuclease-free water was included as a negative control to detect contamination.

PCR amplification was performed under the following thermal cycling conditions: initial denaturation at 95 °C for 30 seconds, 35 cycles of 95 °C for 30 seconds, 60 °C for 30 seconds, 72 °C for 30 seconds, and a final extension at 72 °C for 5 minutes. Amplified products were separated by electrophoresis on a 1% agarose gel and visualized using the GelDoc Go Imaging System (Bio-Rad).

*RNA Extraction and Quantification*

From plants confirmed to carry *Mu* insertions, tissues were collected from two developmental stages: the juvenile stage (V3) and the transition/adult stage (V6). Two inches of leaf tissue from the leaf tips were flash-frozen in liquid nitrogen, ground to a fine powder, and stored in TRIzol reagent (Invitrogen) until extraction. Total RNA was extracted using the Direct-zol™ RNA Miniprep Plus Kit (Zymo Research, R2072), following the manufacturer’s instructions including the DNase I treatment.

RNA quality was initially assessed using the Qubit™ RNA IQ Assay (Invitrogen), and concentration was quantified with the Qubit™ RNA BR Assay Kit (Thermo Fisher Scientific). RNA integrity and quantity were further verified by electrophoresis on a 1.5% agarose gel. Only samples with high RNA integrity were selected for downstream analyses.

*qRT-PCR and Data Analysis*

Reverse transcription and real-time PCR were performed on high-quality RNA samples in a single step using the Luna® Universal One-Step RT-qPCR Kit (E3005X; New England Biolabs), following the manufacturer’s protocol. Reactions were carried out on the CFX Connect Real-Time PCR Detection System (Bio-Rad).

Transcript levels of three genes, *AuxRP1* , *Yucca2* , and *TSB2C* , were measured, with *Ubiquitin* used as an internal control. Relative gene expression was calculated using the ΔCt method (Livak and Schmittgen, 2001), with expression levels of each gene normalized to *Ubiquitin* and compared to W22 wildtype at the same developmental stage. For each genotype, two to three biological replicates and three technical replicates were included. Average relative expression values and standard errors were calculated and reported in the figure. Statistical analysis was conducted using the non-parametric Mann-Whitney U test (also known as Wilcoxon Rank Sum Test) to compare ∆Ct between insertion and wildtype groups at each time point (Chen et al., 2006).

## Reagents

*Primer Sequences*

**Table:** 

**Name**	**Forward**	**Reverse**
**Genotyping PCR**		
*JZMB + JGp3* (For genotyping AcDs-00676)	5’-GCGTCCAAGCCTCAACAGGGTC-3’	5’ -ACCCGACCGGATCGTATCGG-3’
*JZMB +TIR6* (For genotyping UFMu-08200)	5’-GCGTCCAAGCCTCAACAGGGTC-3’	5’-AGAGA- AGCCAACGCCAWCGCCTCYATTTCGTC-3’
*UF414-1 + TIR6* (For genotyping UFMu-00414)	5’-AATGCGACACTGCTCCTTGT-3’	5’-AGAGA- AGCCAACGCCAWCGCCTCYATTTCGTC-3’
*UF3429-1 + TIR6* (For genotyping UFMu-03429)	5’-CTGATCACCGGGACACTGAC-3’	5’-AGAGA- AGCCAACGCCAWCGCCTCYATTTCGTC-3’
*Tubulin* (Housekeeping Gene for positive control)	5’ CTACCTCACGGCATCTGCTATGT 3’	5’ GTCACACACACTCGACTTCACG 3’
**qRT PCR**		
*AuxRP1*	5’-CTCCTGTTCTCTTCCCGTCG-3’	5’-CTCGAGGTCAAACGGCAGTA-3’
*Yucca2*	5’-CGGACGCACTCTTGACTTC-3’	5’-AAGGAATCGTTGCTGCTCTC-3’
*TSB2C*	5’-ATGTGGAGACCACACACTATATC-3’	5’-CGGGTTTCCTTGCCAATAAC- 3’
*Ubiquitin*	5’-GTCATAGTTCTGGGTAGTACGC-3’	5’-TGGAGGTTGTCAAAGTATCTGC-3’
